# Helen Avon

**Published:** 1884-09

**Authors:** 


HELEN AVON; OR, THE LIFE OF A WOMAN SHADOWED BY DEATH. By J. R. S., "The Special Agent," Emory College, Ga. Jas. P. Harrison & Co., publishers, Atlanta, Ga.

This is a beautiful little story, well written and very readable. It is a perfect gem of typographical neatness.

